# Factors Associated with Differences in Physicians’ Attitudes toward Percutaneous Endoscopic Gastrostomy Feeding in Older Adults Receiving End-of-Life Care in Japan: A Cross-Sectional Study

**DOI:** 10.1089/pmr.2023.0088

**Published:** 2024-07-08

**Authors:** Yoko Sakamoto, Toshiharu Mitsuhashi, Katsuyuki Hotta

**Affiliations:** Center for Innovative Clinical Medicine, Okayama University Hospital, Okayama, Japan.

**Keywords:** attitude, end-of-life care, older persons, decision making, percutaneous endoscopic gastrostomy, tube feeding

## Abstract

**Background:**

Although percutaneous endoscopic gastrostomy (PEG) placement is still widely practiced in Japan, studies from Western countries report that it is less beneficial for patients in end-of-life care with cognitive decline. Decisions regarding PEG placement are largely influenced by physician judgment.

**Objectives:**

The aim of this study was to investigate the background and perceptions of Japanese physicians regarding PEG for older adults in end-of-life care and to identify the factors associated with differences in physician judgment regarding PEG.

**Design:**

The study employed a cross-sectional design.

**Setting/Subjects:**

A questionnaire on PEG for older adults in end-of-life care was sent to Japanese physicians. Logistic regression analysis was used to calculate the odds ratios (ORs) and confidence intervals (CIs) of the association between PEG recommendations and each factor.

**Results:**

PEG placement was advised for bedridden patients and older adults with cognitive decline by 26% of the physicians who responded to the survey. Differences in physician perceptions of PEG feeding were associated with the recommendation for PEG, benefits of preventing aspiration pneumonia (OR: 4.9; 95% CI: 3.1–8.2), impact on post-discharge accommodation decisions (OR: 6.1; 95% CI: 1.9–30.9), and hesitancy to recommend a PEG placement (OR: 1.9; 95% CI: 1.3–4.5). Working in a facility with PEG placement (OR: 2.0; 95% CI: 1.2–3.5) was an associated background factor.

**Conclusions:**

Differences in Japanese physicians’ attitudes toward using PEG feeding for older adults in end-of-life care were significantly associated with differences in their perceptions of the impact of PEG feeding and working in a facility with PEG placement.

## Key Message

Differences in Japanese physicians’ perceptions on recommending or withholding percutaneous endoscopic gastrostomy (PEG) for older adults in end-of-life care were linked to factors such as aspiration pneumonia prevention, post-discharge accommodations, hesitancy to recommend a PEG placement, and working in a PEG-providing facility. Improved communication processes are crucial for decision-making regarding PEG.

## Introduction

Numerous reports from Western countries have shown that percutaneous endoscopic gastrostomy (PEG) feeding in older adults in end-of-life care with cognitive decline is neither beneficial^1–4^ nor recommended by current guidelines.^5,6^ However, a higher proportion of older adults undergo PEG placement in Japan than in Western countries.^7,8^ Thus, the use of PEG feeding in older adults with advanced dementia and age-related cognitive decline has generated extensive debate. In 2018, the Japanese Ministry of Health, Labor, and Welfare established the “Guidelines for the Decision-Making Process of Healthcare for Elderly People in the Last Phase of Life”;^9^ in response, other related academic societies likewise developed their own guidelines.^10^ With this development, although the number of older adults in end-of-life care receiving PEG has declined since its peak in 2007, a high number of patients still undergo the procedure.^11,12^

In Japan, family members and physicians are often aware that older adults with cognitive decline are unable to provide consent independently. Therefore, in the absence of instructions from the individual, family members become proxy decision makers.^13^ In cases where oral intake becomes difficult, the choice to withhold artificial hydration and nutrition (AHN) is ultimately directly linked to death; therefore, decision making is fraught with conflict and may pose a significant burden for the individual and their family.^13^ In Japan, decisions regarding AHN, including PEG feeding, are entrusted to physicians by patients’ families. As family members make decisions by proxy with the support of physicians, the decision on whether to implement or withhold PEG placement is significantly influenced by the information provided by the physician’s judgment.^13,14^ According to the “Survey on Attitudes towards Medical Care in the Last Phase of Life, 2017” published by the Ministry of Health, Labor, and Welfare,^15^ 59.1% of physicians responded that they “do not know,” “do not intend to refer to,” or “do not need” uniform standards or national or academic guidelines for medical care in the last phase of life. Thus, the information provided by physicians on whether to recommend or withhold PEG is based on their preferences and opinions.^16^ Therefore, understanding the backgrounds and experiences that influence physicians’ attitudes toward PEG feeding in older adults is important.

Although previous studies have surveyed physicians with limited medical specialties to determine their attitudes, such as geriatricians certified by the Japanese Geriatrics Society, general surgeons, and neurosurgeons,^16–20^ older adults encounter physicians with a wide range of medical specialties. Additionally, differences in geographic regions, medical institutions, and clinical experience of the physicians may contribute to the differences in perceptions. However, currently, no national cross-sectional surveys exist in Japan on physicians’ perceptions, background, and experiences with PEG placement in older adults.

This study aimed to investigate the background and perceptions of Japanese physicians regarding PEG placement in older adults in end-of-life care as well as the factors that cause differences in physicians’ attitudes.

## Methods

### Participants and survey

A web-based questionnaire survey was conducted with approximately 3,000 physicians working at medical institutions (including clinics) and geriatric health care facilities nationwide using the Internet. As an incentive, all respondents were provided with an Amazon gift card worth JPY 1,000 (equivalent to approximately USD 9). A research company (Cando Corporation, Osaka, Japan) was commissioned to select participants and conduct the survey. Email invitations for participation were sent randomly from the database of physicians nationwide registered with the research company. Pediatricians and obstetricians were excluded because they were not involved in caring for older adults. The collected data were anonymized before being shared with the researchers. The survey was terminated when the expected number of participants was reached (*n* = 520). The study period was 3 months, from April to July 2020.

### Questionnaire

A case study on the placement of a PEG device in end-of-life care was presented, and the respondents were requested to answer questions from a list of options. A fictitious case scenario was developed as a reference to a previous study^19^ (Appendix 1). The participants were inquired about the recommendation for PEG and their perceptions of its impact (four items: intention to recommend or withhold PEG, benefits in preventing aspiration pneumonia, decision on where to stay after discharge, contribution to improvement in quality of life, and hesitancy to recommend a PEG placement) on the patient (Mr. A) in this fictitious case. Other information collected included respondents’ demographics, information on medical facilities, experience as a physician, information on views, and whether they were aware of various societal guidelines for end-of-life care. The response options for the questions on views and perceptions were as follows: (1) very much agree, (2) somewhat agree, (3) slightly agree, (4) do not agree at all, and (5) do not know.

Responses (1) and (2) were both considered “agree” and (3), (4), and (5) as “disagree,” which were applied in the analysis as binary variables.

### Statistical analyses

First, descriptive statistics were calculated for participant characteristics and background factors. Continuous variables were described as means and standard deviations, and categorical variables were described as frequencies and percentages. Next, the number and percentage of respondents were calculated for the five items of physicians’ perceptions of PEG feeding, and a binomial test was conducted to determine the differences between the positive and nonpositive response groups for each response. In addition, logistic regression analysis was performed using the recommendation for PEG as the dependent variable and the four items of physicians’ perceptions of the impact of PEG as the independent variables. In addition, penalized maximum likelihood logistic regression was performed with age, sex, department, type of health care facility, practice area, and experience as a primary care physician for older adults as independent variables. Finally, multivariable logistic regression analysis was performed with physicians who reported that they had experience as primary care physicians for the older adults, with the health care facility where PEG was performed, experience as a PEG surgeon, and familiarity with relevant guidelines as independent variables. Odds ratios (ORs) and 95% confidence intervals (CIs) were calculated for all analyses. The significance level was set at two-sided α = 0.05. The statistical software Stata/SE16 (StataCorp, College Station, TX, USA) was used for all the analyses.

### Ethics approval and consent to participate

A consent form was posted online at the beginning of the questionnaire, and only those who provided consent were allowed to complete the questionnaire. This study was approved by the Ethics Review Committee of Okayama University (approval no.: K2004-005).

## Results

The survey was conducted with 3,078 physicians in Japan, of which a total of 564 responded (18.3% response rate). Although the target sample size was 520, the nationwide nature of the survey caused the number of responses to exceed the planned target owing to budgetary constraints. The survey was designed such that no unselected responses could be sent, to minimize the dropout data, and all 564 respondents were included in the analysis.

The majority of the respondents were male physicians, and there were a small number of female physicians. Half of the respondents were 50–69 years of age, whereas some were 70 years of age or older. All 12 physicians working at the geriatric facility were over 70 years of age (Table 1).

**Table 1.** Respondents’ Demographic Characteristics

Characteristic	*n* %
	564 (100.0)
Sex	
Male	531 (94.1)
Female	33 (5.9)
Age mean (SD)	51.5 (9.4)
Age category (years)	
24–39	69 (12.2)
40–49	168 (29.8)
50–59	204 (36.2)
60–69	111 (19.7)
70 and over	12 (2.1)
Department	
Internal Medicine	362 (64.2)
Surgery	157 (27.8)
Emergency	27 (4.8)
Other	18 (3.2)
Primary practice setting	
University Hospital	124 (22.0)
More than 200 hospital beds	234 (41.5)
Less than 200 hospital beds	90 (16.0)
Clinic (only outpatient)	104 (18.4)
Health care facilities for older adults	12 (2.1)
Regional Classification	
Hokkaido and Tohoku Region	28 (5.0)
Kanto Region	151 (26.8)
Tokyo Metropolis	130 (23.0)
Kansai Region	143 (25.4)
Chubu Region	59 (10.5)
Chugoku/Shikoku Region	28 (5.0)
Kyushu/Okinawa Region	25 (4.4)

**FIG. 1.**
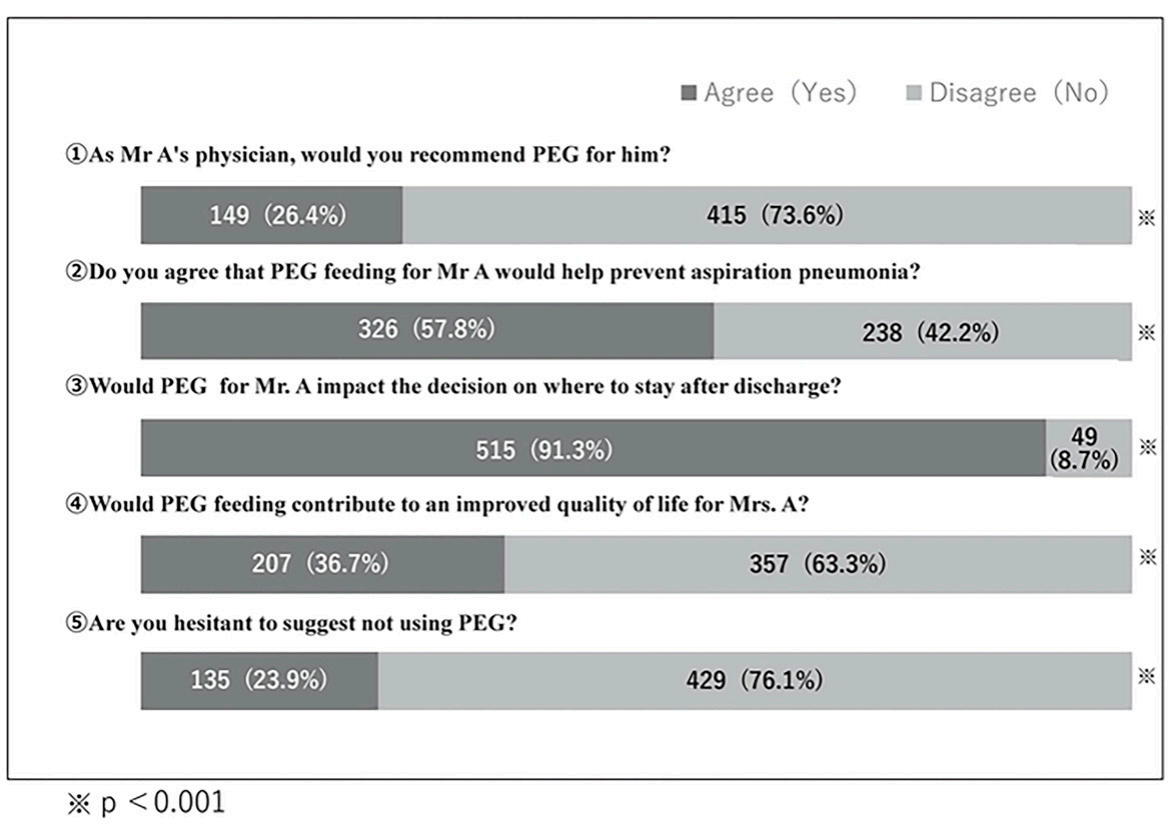
The number of responses on physicians’ perceptions of PEG in older adults in end-of-life care PEG: percutaneous endoscopic gastrostomy.

As shown in Figure 1, 26.4% of the physicians recommended PEG for older adults. Nearly half of the physicians were divided based on their perception of whether PEG feeding was beneficial in preventing aspiration pneumonia. Most physicians (91.3%) agreed that PEG placement would affect the decision on where to discharge the patient, and 36.7% agreed that PEG feeding would benefit the quality of life. One-quarter of the physicians were reluctant to recommend withholding PEG placement at the time of decision-making. Significant differences were observed between “agree” and “disagree” responses for all five items (*p* < 0.001).

The results of the univariate logistic regression analysis are shown in Table 2, with each of the four perceptions of the impact of using a PEG tube as the independent variable, the recommendation or withholding of PEG placement as the dependent variable, and the number and percentage of recommendations for each variable. The OR for PEG feeding to prevent aspiration pneumonia was 4.9 (95% CI: 3.1–8.2); this was higher for those who recommended it than for those who did not. The OR for recommending PEG placement was 6.1 (95% CI: 1.9–30.9) for those who said that PEG placement would impact the patient’s decision on where to discharge. The OR for those who thought it would help improve patients’ quality of life was 15.6 (95% CI: 9.5–25.8), and that for those who were hesitant to recommend a PEG placement was 1.9 (95% CI: 1.3–4.5). The OR for recommending PEG placement was significantly higher for each variable.

**Table 2.** Physicians’ Perceptions of Percutaneous Endoscopic Gastrostomy and Odds Ratio for Their Percutaneous Endoscopic Gastrostomy Recommendations or Withholding (*n* = 564)

Physicians’ perceptions of PEG	Positive response group	Negative response group	OR (95% CI)
	No. of PEG recommendations/total no. (%)		
Benefit of Aspiration Pneumonia Prevention	123/326 (37.7)	26/238 (10.9)	4.9 (3.1–8.2)
Determining where to stay after discharge	146/515 (28.3)	3/49 (6.1)	6.1 (1.9–30.9)
Contribution to quality of life improvement	120/207 (60.0)	29/357 (8.1)	15.6 (9.5–25.8)
Hesitation to recommend withholding	59/135 (43.7)	90/429 (21.0)	1.9 (1.3–4.5)

CI, confidence interval; OR, odds ratio; PEG, percutaneous endoscopic gastrostomy.

The number of PEG recommendations for each background factor and the results of the penalized maximum likelihood logistic regression with background factors as independent variables are presented in Table 3. The point estimates of both the ORs were low at 0.2, as no physician 70 years of age or older, as well as those working in geriatric facilities recommended PEG placement. For the variable of regional classification, physicians in rural areas had a lower OR for recommending PEG placement than those in Tokyo (a significant difference was observed only in the Chūbu region [OR: 0.4; 95% CI: 0.2–0.8], with no significant differences in other regions). For the variable of physician experience, none of the differences in the recommendation of PEG placement according to experience were statistically significant. In the description of relevant guidelines, 56 physicians did not refer to any relevant guidelines, and nearly 40% of all physicians did not refer to any guidelines, including 155 who stated that they were not informed of the guidelines. The OR for recommending PEG was lower for physicians who did not refer to or were not aware of the guidelines compared with that for those who referred to the guidelines ([OR: 0.8; 95% CI: 0.4–1.6] and [OR: 0.9; 95% CI: 0.5–1.4], respectively).

**Table 3.** Association of Physician Background and Percutaneous Endoscopic Gastrostomy Recommendation Analyzed by Penalized Maximum Likelihood Logistic Regression (*n* = 564)

Physician backgrounds	No. of PEG recommendations/ total no (%)	OR (95% CI)
Sex		
Male	142/531 (26.7)	
Female	7/33 (21.2)	0.7 (0.3–1.7)
Age category		
20–39	18/69 (26.1)	Reference
40–49	39/168 (23.2)	0.9 (0.4–1.8)
50–59	66/204 (32.4)	1.4 (0.7–3.0)
60–69	26/111 (23.4)	0.9 (0.4–2.0)
70 and over	0/12 (0.0)	0.2 (0.0–3.7)
Department		
Internal Medicine	91/362 (25.1)	Reference
Surgery	48/157 (30.6)	1.2 (0.8–1.9)
Emergency	7/27 (25.9)	0.9 (0.4–2.4)
Other	3/18 (16.7)	0.7 (0.2–2.3)
Primary practice setting		
University Hospital	32/124 (25.8)	Reference
More than 200 hospital beds	24/90 (26.7)	1.4 (0.8–2.4)
Less than 200 hospital beds	73/234 (31.2)	1.2 (0.6–2.3)
Clinic (only outpatient)	20/104 (19.2)	0.8 (0.4–1.5)
Health care facilities for the elderly	0/12 (0.0)	0.2 (0.0–4.0)
Regional Classification		
Tokyo Metropolis	40/130 (30.8)	Reference
Hokkaido and Tohoku Region	7/28 (25.0)	0.7 (0.3–1.8)
Kanto Region	39/151 (25.8)	0.7 (0.4–1.3)
Kansai Region	44/143 (30.8)	1.0 (0.6–1.6)
Chubu Region	9/59 (15.3)	0.4 (0.2–0.8)
Chugoku/Shikoku Region	6/28 (21.4)	0.7 (0.2–1.9)
Kyushu/Okinawa Region	4/25 (16.0)	0.4 (0.1–1.2)
Experience as attending Physician for elderly patients		
No	17/61 (27.9)	
Yes	132/503 (26.2)	1.1 (0.6–2.0)
Experience of Home Health Care Physicians		
No	108/389 (28.8)	
Yes	41/175 (23.4)	1.0 (0.6–1.5)
Experience of Community health care learning program		
No	114/426 (26.8)	
Yes	35/138 (25.4)	1.1 (0.6–1.9)
Involvement in Community Comprehensive Care		
No	132/481 (27.4)	
Yes	17/83 (20.5)	0.7 (0.4–1.3)
Awareness of relevant guidelines		
Use as reference	97/353 (27.5)	Reference
Not use as reference	13/56 (23.2)	0.8 (0.4–1.6)
Don’t know the guidelines	39/155 (25.2)	0.9 (0.5–1.4)

CI, confidence interval; OR, odds ratio; PEG, percutaneous endoscopic gastrostomy.

Table 4 shows the results of the multivariable logistic regression analysis among 503 physicians with experience with geriatric populations, with each variable related to practice experience as an independent variable. The OR for PEG recommendation was 2.0 (95% CI: 1.2–3.5) for physicians working in facilities where PEG was performed, which was considerably higher than the corresponding figure from facilities where it was not performed. The OR of recommendation for physicians with surgical experience was 1.4 (95% CI: 0.9–2.2).

**Table 4.** Association between Practice Experience and Percutaneous Endoscopic Gastrostomy Recommendation among Physicians Who Had Experience with Older Persons Analyzed by Multivariable Logistic Regression (*n* = 503)

	No. of PEG recommendations/ total no (%)	OR (95% CI)
Number of patients to contact		
≤50	76/273 (27.8)	Reference
50 < 100	22/97 (22.7)	0.7 (0.4–1.3)
≥100	34/133 (25.6)	0.8 (0.5–1.3)
Frequency of contact		
< 3 days/1 month	63/243 (25.9)	Reference
< 2 days/1 week	9/49 (18.4)	0.5 (0.2–1.2)
≤3 days/1 week	60/211 (28.4)	0.8 (0.5–1.3)
Work place is a PEG facility		
Yes	109/364 (29.9)	2.0 (1.2–3.5)
Experienced in surgeries for PEG		
Yes	48/144 (33.3)	1.4 (0.9–2.2)

CI, confidence interval; OR, odds ratio;  PEG, percutaneous endoscopic gastrostomy; ref, reference.

## Discussion

This study investigated the background and perceptions of Japanese physicians regarding decision making for recommending PEG for geriatric populations in the last stage of life. It also identified factors associated with differences in physicians’ attitudes toward PEG. The results showed that one-quarter of the participating physicians recommended PEG device placement for bedridden older adults in end-of-life care with cognitive decline. Physician perceptions of PEG feeding (benefits of preventing aspiration pneumonia, impact on decisions regarding where to stay after discharge, improved quality of life, and hesitancy to recommend a PEG placement) were significantly associated with their recommendations for PEG placement. Background factors that contributed to differences in decision-making of physicians were identified, including working in a facility where PEG was performed.

This study found nearly half of its respondents to agree that the use of PEG was beneficial for preventing aspiration pneumonia in older adults in end-of-life care and that these physicians were more likely to recommend PEG placement. Although many previous reports have shown that the use of PEG does not prevent aspiration pneumonia,^4–6,21,22^ the views of many physicians were discrepant.^18,23,24^ In addition, it was found that approximately 40% of the physicians did not refer to the guidelines, although these results were consistent with previous studies showing that discrepancies in views on not benefiting the prevention of aspiration pneumonia were associated with not referring to the guidelines.^18,23^ Previous studies have highlighted the insufficient guidelines on PEG in end-of-life care, such as for older adults with advanced dementia, and have suggested the need for guidelines to be developed.^17,25^ Therefore, it is disconcerting that despite the publication of these guidelines, they have not been referenced. Further information and education regarding these guidelines are needed in the future.

Most physicians perceived that PEG placement affected the decision on where to discharge, and the physicians’ decision to recommend PEG placement may be more reflective of the family’s desires. According to a previous study in the United States, 63% of the families requested PEG tube placement even when physicians did not recommend it.^26^ In Japan, nursing home staff often prefer PEG feeding to time-consuming oral nutrition to facilitate care.^27,28^ Therefore, family preference for admission to a care facility may have influenced their decision making.^29^ However, a study on family’s decision-making regret reported that the subsequent regret was greater when the patient’s wishes were not confirmed.^29^ It is very important to ascertain the patient’s preference before the end of life, and advance care planning should be more widely used.^30,31^

Two contradictions exist in the results of this study. First, several physicians responded that they had no hesitation in withholding PEG tube placement, and 21% of them shared that they would recommend it. Second, although there was no significant difference, a higher proportion of physicians who were not aware of the relevant guidelines stated they would not recommend PEG tube placement than those who were aware of the guidelines. This indicates that the awareness of the relevant guidelines did not influence the recommendation for PEG. These discrepancies suggest that there could be justifications for favoring the recommendations. As mentioned above, family wishes or admission to a care facility may be relevant reasons.^26–29^ Alternatively, indications/contraindications for PEG placement are a separate issue from consent to PEG placement.^18^ Although previous studies have shown that physician decisions have a significant influence on families,^14,16^ the results of this study suggest that family’s wishes may also influence physicians’ decisions.^16,26,29^ In other words, effective communication between the two entities is more important, as they may influence each other’s thought processes.

Our study yielded the following important findings: physicians working in PEG-providing facilities were considerably more likely to recommend PEG placement (OR: 2.0; 95% CI: 1.2–3.5). Furthermore, physicians with previous surgical experience for PEG were more likely to recommend PEG placement (OR: 1.4; 95% CI: 0.9–2.2).

No previous studies have previously investigated these two factors for PEG, although they have described differences in decision making for PEG placement between departments and physicians’ years of experience. This is presumably because of the ease of management after PEG; further investigation is required because the questionnaire did not investigate this reason.

This study has some limitations. First, owing to the male-biased sex ratios of the participants of the study, the generalization of the results should be cautioned against. Second, although the results showed no significant differences according to department, region, medical institution, or experience with community education or home care; there may have been insufficient statistical power owing to the limited size of the participant sample. Additional large-scale studies are needed to identify differences in these factors. Third, the response rate for the web-based questionnaire was low (18.3%). However, the actual response rate may have been higher than the figures presented because the number of people to whom the survey was distributed was calculated as the total number of people, and the survey may have been distributed multiple times to the same physician. There is a concern that differences in the Internet environment and the frequency of physicians checking their emails may have affected the selection of the target group. Physicians who responded to the questionnaire possibly represented a group that was more interested in providing decision-making support for end-of-life care. Therefore, their awareness regarding these guidelines may be higher compared with the general population. Fourth, as this was an exploratory cross-sectional study, the causal effects were not identified. Therefore, these results should be cautiously interpreted. Furthermore, this survey did not include questions to confirm patient or family preferences regarding PEG placement. Future studies are required to limit and validate the factors that explain physicians’ attitudes.

In conclusion, this study showed that differences in physicians’ attitudes toward using PEG feeding among older adults in end-of-life care were notably associated with differences in their perceptions of the impact of PEG feeding and working in a facility with PEG placement. Further discussion and consideration of the communication process among physicians, patients, and their families is needed for decision making regarding PEG tube placement for geriatric populations in end-of-life care.

## Abbreviations Used

AHN: Artificial Hydration and Nutrition
CI: Confidence Interval
JPY: Japanese Yen
OR: Odds Ratio
PEG: Percutaneous Endoscopic Gastrostomy
SD: Standard Deviation
SE: Standard Error
USD: United States Dollar

## Appendix 1

[Case]

Mr. A, a 90-year-old man, resides with his 78-year-old wife in their home. He experienced a stroke 6 months previously and is currently hospitalized. He presented with right-sided paralysis and is currently undergoing rehabilitation. However, his activities of daily living gradually declined, and he became mostly bedridden. In addition, his cognitive function declined, resulting in difficulties with speech and hearing.

Later, Mr. A exhibited a reluctance to eat and received dysphagia rehabilitation with a speech–language–hearing therapist. Despite these interventions, his oral intake declined to approximately 10% of normal. With his upcoming discharge from the hospital, his wife was faced with the difficult decision of whether to transfer him to a nursing home or opt for home care services for him. Although his wife is the primary decision maker, his eldest daughter, who lives separately, recommended nursing home care.
